# The Climate Change Worry Scale (CCWS) and Its Links with Demographics and Mental Health Outcomes in a Polish Sample

**DOI:** 10.3390/healthcare12111128

**Published:** 2024-05-31

**Authors:** Paweł Larionow, Magdalena Gawrych, Julia Mackiewicz, Maciej Michalak, Karolina Mudło-Głagolska, David A. Preece, Alan E. Stewart

**Affiliations:** 1 Faculty of Psychology, Kazimierz Wielki University, 85-064 Bydgoszcz, Poland; 2 Institute of Psychology, The Maria Grzegorzewska University, 02-353 Warsaw, Poland; 3 Faculty of Health Sciences, School of Population Health, Curtin University, Perth, WA 6102, Australia; 4 School of Psychological Science, The University of Western Australia, Perth, WA 6009, Australia; 5 Department of Psychology, The School of Humanities and Sciences, Stanford University, Stanford, CA 94305, USA; 6 Department of Counseling and Human Development Services, The University of Georgia, Athens, GA 30677, USA; 7 Department of Geography, The University of Georgia, Athens, GA 30677, USA

**Keywords:** climate anxiety, climate change worry, eco-anxiety, eco-emotions, environmental distress, experience of climate change, mental health, pro-environmental behavior, psychometric properties, solastalgia

## Abstract

Developing valid and reliable measures of psychological responses to climate change is of high importance, as this facilitates our understanding of people’s psychological responses, including their pro-environmental behavior. Recently, the Climate Change Worry Scale (CCWS) was introduced. This study aimed to develop the first Polish version of the CCWS and explore its psychometric properties. Our sample comprised 420 Polish adults aged 18–70, with a mean age of 26.20 (standard deviation = 10.61) years. The CCWS’s factor structure was assessed with confirmatory factor analysis. McDonald’s omega and Cronbach’s alpha coefficients were computed to assess internal consistency reliability. Pearson correlations between climate change worry (CCW) and experience of climate change (i.e., an individual’s level of perception of being affected by climate change), pro-environmental behavior, ill-being (i.e., anxiety and depression symptoms), and well-being were calculated. Our results support the strong factorial validity of the CCWS, conforming to its intended one-factor solution, with excellent internal consistency reliability for the total scale score (i.e., McDonald’s omega and Cronbach’s alpha values of 0.93). We noted large positive correlations between CCW and experiences of climate change, as well as pro-environmental behavior, and medium positive correlations with psychopathology symptoms. CCW scores were not associated with well-being. As the CCWS represents a measure of a specific manifestation of worry, we also examined its discriminant validity against more general psychological distress markers, and it evidenced strong validity in this regard. Overall, the Polish version of the CCWS appears to have strong psychometric properties, and will therefore be a useful tool to use in research on psychological responses to climate change.

## 1. Introduction

Climate change and people’s perceptions of this phenomenon are associated with a wide range of psychological responses, including various phenomena termed climate anxiety [1], climate emotions [2], and climate change worry (CCW) [3,4]. While climate anxiety represents a negative psychological state expressed in emotional (e.g., irritability, fear), cognitive (e.g., difficulty concentrating), physiological (e.g., nausea, sweating), and behavioral (e.g., sleep disturbances) indicators [5], climate emotions can be classified according to their valence. For example, researchers distinguish negative emotions (e.g., anger, sorrow, contempt, guilt, climate hopelessness) from positive emotions (e.g., climate hopefulness, climate empowerment, and others) stemming from perceptions of climate change [6]. In contrast, CCW refers to ruminating thoughts regarding climate change and its consequences [3], with no explicitly expressed emotional (e.g., nervousness), physiological (e.g., muscle tension), or behavioral (e.g., impairment in daily functioning) indicators, which are more commonly associated with climate anxiety [5]. CCW can be considered as a verbal–linguistic process of producing ruminative thoughts (e.g., rumination and worry about the climate) [3]. According to Ojala et al. [7], the nature of CCW seems to be more cognitive than the nature of climate anxiety; CCW acts as a first step in coping efforts, whereas climate anxiety can be considered as an outcome of CCW. Based on these considerations, CCW is of interest for use as a construct to learn more about the process of developing psychological responses to climate change.

One promising new measure for assessing CCW is the Climate Change Worry Scale (CCWS), which was originally developed in English by Stewart [3]. The CCWS is a ten-item tool for assessing disturbing thoughts related to climate change (e.g., “Thoughts about climate change cause me to have worries about what the future may hold”; “I worry that outbreaks of severe weather may be the result of a changing climate”) [3]. Items are answered on a 5-point response scale from 1 (“never”) to 5 (“always”), indicating the frequency of the experience of climate change-related worries, with higher scores indicating more worries [3]. All items are designed to be summed into a total score (i.e., a one-factor solution). The current study aimed at introducing the first Polish version of the CCWS, and examining its psychometric properties.

In terms of existing psychometric data, the original English CCWS [3] showed good psychometric properties, including the strong factorial validity of the intended one-factor solution, excellent internal consistency reliability (i.e., McDonald’s omega and Cronbach’s alpha values were ≥0.90), and good convergent and divergent validity. CCWS scores were moderately positively correlated with general anxiety and depression symptoms, with statistically significant correlations around 0.30 [3]. In contrast, CCWS scores were negatively correlated with well-being, with statistically significant correlations around −0.20 [8]. Slovenian [8] and Italian [9] versions have also been tested, broadly supporting the validity and reliability of the scale cross-culturally. For example, climate change worry was strongly positively associated with engagement in pro-environmental behaviors (with a statistically significant correlation of 0.52) in the Slovenian version of the CCWS [8]. In the Italian version, a one-factor structure was supported, though those researchers removed two of the items due to low communality values [9].

For the present study, based on theory and the above-described studies, we expected that the subjective experience of climate change (i.e., the level of an individual’s perception of being affected by climate change or awareness of the presence of climate change) would be at least moderately correlated with CCWS scores, as awareness of the presence of climate change seems to be a required factor for experiencing worries about climate change. As for demographic differences, previous studies have shown that CCWS scores were higher in females than in males [3], and age and education level were not statistically significantly associated with education level [8]. We were also interested in examining the differences in CCWS scores with respect to residence, and due to a lack of previous studies on this demographic variable, we had no specific hypothesis. Overall, considering all the above-described results, we anticipated similar outcomes in our study.

In order to facilitate our understanding of psychological responses to climate change in different cultures, we believed that developing the Polish version of the CCWS would make a good contribution to the field. Hence, this paper aimed to introduce and examine the psychometric performance of the first Polish version of the CCWS, and use the tool to explore the nature of the climate change worry construct.

## 2. Materials and Methods

### 2.1. Procedure

This research was conducted according to the Declaration of Helsinki Ethical Principles. The Maria Grzegorzewska University Ethics Committee approved the current study (No. 166/2024). This was a cross-sectional study, which was conducted online via Google Forms from January to February in 2024. A link to this survey was posted on social network pages (i.e., Facebook and Instagram), where we invited Polish-speaking adults aged 18 or above to participate in this anonymous and voluntary study. Participants of 18 years of age or above, and who completed a written informed consent form and successfully passed an attention check, were included in the data analysis. The research data were archived securely, and will be available for five years.

For assessing test–retest reliability, we used a study with a paper-and-pencil format. We invited participants from the convenience sample (i.e., university students) to fill out the CCWS twice, within an approximate two-week interval. This was an anonymous and voluntary study, with unique codes, which were provided to our participants at the first measurement. These codes were used by our participants at the second measurement, and this made it possible to connect anonymized surveys filled in by the same participants between the two measurements.

### 2.2. Participants

Our sample included 420 Polish adults aged 18–70, with a mean age of 26.20 years. The majority of participants were females, inhabitants of large cities, and people with secondary education. Detailed demographic characteristics are presented in Table 1.

### 2.3. Measures

In this study, we used a demographic questionnaire and a set of short self-report measures. The order of the questionnaires used was the same for all participants, starting with a demographic questionnaire and our main measure, the CCWS, and continuing with the other questionnaires in accordance with the order in which they are described below.

1. The *Climate Change Worry Scale* (CCWS) [3] is a self-report measure for assessing the level of ruminating thoughts that people experience about climate change. The scale consists of ten items (e.g., “I worry about climate change more than other people”; “I worry that I might not be able to cope with climate change”), with a five-point Likert scale from 1 (“never”) to 5 (“always”), and a possible range from 10 to 50 [3]. There are no reverse-scored items. A higher score, calculated by summing all the items, indicates a higher level of worry experienced due to climate change [3].

In order to prepare the Polish version of the CCWS, we followed standard translation procedures [10], and invited three independent translators (both fluent in Polish and English, with experience in environmental and health psychology) who translated the original English version of the CCWS into Polish. Based on these translations, we developed a common Polish translation. In order to check the accuracy of this Polish translation against the original version, the authors of this study back-translated this Polish translation into English, and compared this back-translation with the original English version of the CCWS. We analyzed potential discrepancies within this comparison, and provided necessary minor corrections, which resulted in the prefinal Polish version of the CCWS. Before carrying out our psychometric study within a population sample, this prefinal version of the CCWS was evaluated by ten people from a Polish community sample with different demographic backgrounds (age, gender, and education categories), and no significant suggestions for the scale’s improvement were received. Hence, during these procedures, the final version of the CCWS was developed (see Supplementary Materials).

2. The *Experience of Climate Change Scale* (ECCS; original version [1]; Polish version [11]) is a brief measure for assessing an individual’s perception of being affected by climate change. The scale consists of three items (e.g., “I have been directly affected by climate change”; “I have noticed a change in a place that is important to me due to climate change”), with a five-point Likert scale from 1 (“strongly disagree”) to 5 (“strongly agree”), and a possible range from 3 to 15 [1]. The ECCS has no reverse-scored items, and a higher score indicates a higher level of subjective experience of climate change.

3. The *Behavioural Engagement Scale* (BES; original version [1]; Polish version [11]) is a brief measure consisting of six items concerning behavioral practices within environmental conservation efforts (e.g., “I turn off lights”; “I try to reduce my behaviors that contribute to climate change”), with a five-point Likert scale from 1 (“strongly disagree”) to 5 (“strongly agree”), and a possible range from 6 to 30 [1]. The BES has no reverse-scored items, and a higher score indicates a higher level of these behavioral practices. Both the ECCS and the BES item sets were part of Clayton and Karazsia’s original efforts to examine and measure climate anxiety and its correlates [1].

4. The *Patient Health Questionnaire-4* (PHQ-4; original version [12]; Polish version [13]) is a four-item brief measure for detecting anxiety and depressive symptoms experienced in the previous two weeks. The questionnaire consists of two subscales, a two-item anxiety subscale (e.g., “Feeling nervous, anxious, or on edge”), and a two-item depression subscale (e.g., “Feeling down, depressed, or hopeless”) [12]. The PHQ-4 uses a four-point Likert scale from 0 (“not at all”) to 3 (“nearly every day”), with a possible range of 0 to 3 for each subscale. There are no reverse-scored items. The total score can be calculated by adding together the scores of the two subscales, with a possible range from 0 to 12. Higher subscale and total scores indicate higher levels of symptoms [12].

5. The *World Health Organization–Five Well-Being Index* (WHO-5; original version [14], Polish version [15,16]) is a short self-report questionnaire for measuring individual’s current mental well-being. The WHO-5 consists of five items (e.g., “I have felt active and vigorous”; “I woke up feeling fresh and rested”), with a six-point Likert scale from 0 (“at no time”) to 5 (“all of the time”) [14]. There are no reverse-scored items. The scale score is calculated by totaling the figures of the five responses. It ranges from 0 to 25, with higher scores indicating higher well-being [14].

### 2.4. Statistical Analysis

Descriptive statistics and all other analyses, except confirmatory factor analysis (CFA), were computed with *JASP* v. 0.18.3. Factor structure was verified with CFA using a robust version of an unweighted least squares (ULSM) estimator in the *lavaan* package in *R* v. 4.3.1. Comparative fit index (CFI) and Tucker–Lewis index (TLI), with acceptable values of ≥0.90, as well as root mean square error of approximation (RMSEA) and standardized root mean square residual (SRMR), with acceptable values of ≤0.08, were used as fit index values for assessing model fit [17].

For assessing internal consistency reliability, we calculated McDonald’s omega [18] and Cronbach’s alpha [19] coefficients with their 95% confidence intervals (95% CI), with coefficients of ≥0.70 regarded as acceptable [20]. In order to assess test–retest reliability, we calculated an intraclass correlation coefficient (two-way random effects, absolute agreement, single rater/measurement) [21] between two measurements of CCWS scores, with an approximately two-week interval between the measurements. For this intraclass correlation coefficient, 95% CIs were computed.

In order to compare CCWS scores between females and males, a *t*-test with Cohen’s *d* as its effect size estimate was used. Spearman correlations between CCWS scores and age, education, and residence were calculated. Pearson correlation between CCWS scores and other questionnaire scores were computed. Correlation coefficients of 0.12, 0.24, and 0.41 were judged as small, medium, and large [22].

For assessing the discriminant validity of the CCWS against mental health symptoms, we conducted an exploratory factor analysis (EFA) with principal axis factoring and oblimin rotation. In order to determine the number of factors to extract, we used eigenvalues of >1 as the criterion. We input all item scores of the CCWS and PHQ-4 into this analysis. We expected that two factors would be extracted, with a ”CCW” factor composed of CCWS items, and a “general distress” factor composed of PHQ-4 items. In other words, we predicted that CCWS items would be statistically separable from general anxiety and depression symptoms, as measured by PHQ-4 items.

We were also interested in examining the underlying structure and separability of the studied variables. Therefore, we also conducted a broader second-order EFA with principal axis factoring with oblimin rotation. In order to determine the number of factors to extract, we used eigenvalues of >1 as the criterion. In our factor analyses, item factor loadings ≥0.40 were considered as salient loadings [23]. We input all subscale scores (i.e., CCWS, ECCS, BES, PHQ-4 Anxiety, PHQ-4 Depression, and WHO-5) into this analysis. We had no specific hypotheses regarding how many factors would be extracted, as this analysis was explorative.

## 3. Results

### 3.1. CFA

The one-factor original solution of the CCWS showed an excellent fit to the data (χ^2^/df = 143.63/35; CFI = 0.997; TLI = 0.996; RMSEA = 0.043 [90% CI: 0.036; 0.050]; SRMR = 0.044). All item factor loadings loaded strongly on the general CCW factor (see Table 2), with loadings ranging from 0.48 (item 6) to 0.87 (item 4).

### 3.2. Descriptive Statistics and Internal Consistency Reliability

Descriptive statistics with internal consistency reliability coefficients for all the study variables are reported in Table 3.

The CCWS demonstrated excellent reliability, with McDonald’s omega and Cronbach’s alpha values of 0.93. All other questionnaires showed acceptable to good internal consistency reliability, with McDonald’s omega and/or Cronbach’s alpha coefficients from 0.71 to 0.86.

### 3.3. Test-Retest Reliability

A total of 38 people filled out the CCWS twice, within an approximate two-week interval. In this data-set, the intraclass correlation coefficient for CCWS scores measured between the first and second measurements within this time interval was 0.66 (95% CI: 0.29; 0.83), indicating moderate test–retest reliability (as defined by intraclass correlation coefficient values between 0.50 and 0.75 according to the guidelines by Koo and Li [21]). The Pearson correlation coefficient between these two measurements was 0.73 (*p* < 0.001).

### 3.4. Demographic Differences

We compared CCWS scores between females and males with a t-test and observed that females had higher mean levels of CCW than males, *t*(406) = 3.16, *p* = 0.002, Cohen’s *d* = 0.44. Age was not statistically significantly correlated with CCWS scores (Spearman rho = −0.02, *p* > 0.05). Education level, coded with an ordinal scale from 1 (primary) to 4 (university degree), was not statistically significantly associated with CCWS scores (Spearman rho = 0.04, *p* > 0.05). Residence, coded with an ordinal scale from 1 (village) to 4 (a large city with above 100,000 inhabitants), also was not statistically significantly correlated with CCWS scores (Spearman rho = 0.09, *p* > 0.05).

### 3.5. Concurrent Validity

Pearson correlations between the study variables are presented in Table 4.

CCW was positively correlated with experience of climate change (r = 0.59, *p* < 0.001) and engagement in pro-environmental behaviors (r = 0.51, *p* < 0.001), and positively with anxiety (r = 0.33, *p* < 0.001) and depression symptoms (r = 0.20, *p* < 0.01). There were no statistically significant correlations between CCW and well-being (r = −0.02, *p* > 0.05).

### 3.6. Discriminant Validity

The EFA extracted two factors, with an eigenvalue of 6.56 for Factor 1, and an eigenvalue of 2.42 for Factor 2. Factor 1, which we called “CCW”, was composed of the ten CCWS items, and explained 41.29% of the variance. Factor 2, which we called “general psychological distress”, was composed of four PHQ-4 items and explained 17.36% of the variance. The two factors were positively interrelated (r = 0.30). All factor loadings were strong, and there were no cross-loadings (see Table 5). This EFA suggests that CCWS scores represented a construct that was separable from general psychological distress.

### 3.7. Examining the Underlying Structure of the Studied Variables

The second-order EFA extracted two factors, with an eigenvalue of 2.36 for Factor 1, and an eigenvalue of 1.84 for Factor 2. Factor 1 explained 28.01% of the variance, whereas Factor 2 explained 27.66% of the variance, with these factors accounting for 55.67% of the cumulative variance. All factor loadings were strong, and there were no cross-loadings (see Table 6).

Factor 1 was composed of CCW, experience of climate change, and behavioral engagement subscale scores, which overall represent climate change-related variables. Therefore, we called this Factor 1 “active attitude towards climate change”, with CCWS scores being the core component, having their highest factor loading in this attitude. Factor 2 was composed of anxiety and depression symptoms and well-being scores, with well-being loading negatively on the factor. Therefore, this factor was called “negative mental health outcomes”, with higher scores on this factor indicating worse mental health (i.e., higher anxiety and depression symptoms with lower well-being). Factor 1 and Factor 2 were positively correlated (r = 0.16). These analyses therefore indicate that, in general, climate change-related variables, including CCWS scores, were statistically separable from people’s current level of general mental health outcomes.

## 4. Discussion

In this study, we introduced the Polish version of the CCWS, examined its psychometric properties, and used this tool to learn more about the nature of the CCW construct. Our findings support its intended one-factor structure, excellent internal consistency reliability, and moderate test–retest reliability. Its concurrent validity was good, and a strong discriminant validity against more general mental health outcomes was noted. All these findings support the good psychometric performance of the Polish version of the CCWS.

### 4.1. Factor Structure, Internal Consistency and Test–Retest Reliability

As expected, our CFA supported the intended one-factor structure of the CCWS, with excellent fit indices. This one-factor solution was previously supported in other validation studies [3,8], indicating that all ten CCWS items measure the CCW construct meaningfully. It also suggests good cross-cultural applicability of the scale, as its key psychometric properties were strong in different cultures [3,8]. Our results empirically support that the internal consistency reliability of CCWS scores was excellent (i.e., McDonald’s omega and Cronbach’s alpha values of 0.93), thus supporting the conclusions presented in the English and Slovenian versions of the CCWS [3,8]. As for the Italian version of the scale by Innocenti et al. [9], in that study, two initial CCWS items (items 6 and 7) were removed due to their factor loadings of <0.7. However, this can be considered a strict criterion, as frequently in the psychometric literature, 0.40 is used as a cut-off to indicate a well-performing item factor loading (e.g., [23]). In our Polish study, all CCWS items had good factor loadings of ≥0.40, and the CFA fit indices were excellent, suggesting that no modifications of the scale were needed. The cross-cultural comparability of the different language versions of the scale seems to be good based on current evidence [3,8], and this may encourage researchers towards cultural measurement invariance testing.

Regarding the test–retest reliability of the Polish CCWS, our results indicate that it was moderate in our data-set, with an intraclass correlation coefficient of 0.66 in a sample of 38 people. In contrast, Stewart [3] found a good temporal stability of CCWS scores across a two-week interval, with the Pearson correlation coefficient of r = 0.91 in a sample of 54 people. As we used the same two-week interval, our results (with the Pearson correlation coefficient of r = 0.73) seem to be broadly comparable with Stewart’s ones [3]; however, our test–retest sample (*n* = 38) was moderate in size, and smaller that Stewart’s one. Despite the fact that we supported the moderate test–retest reliability of the Polish CCWS, future studies with larger samples would be beneficial in order to examine the stability over time of the CCW.

### 4.2. Concurrent and Discriminant Validity

As expected, CCW, as measured by the CCWS, was relatively strongly positively associated with one’s perception of being affected by climate change. This relationship is expected, because awareness of climate change seems to be an indispensable condition for the development of CCW. We also noted that higher levels of CCW were associated with higher levels of behavioral practice within environmental conservation efforts, indicating that CCW is not an isolated psychological phenomenon. In our correlational analysis, CCW, whilst being associated with other climate-related variables and psychopathology symptoms (in line with our expectations), was not associated with well-being scores. As previous studies noted a negative correlation between CCWS scores and well-being indicators [8], a lack of this correlation in our study was something of an unexpected result. It might be explained by sample characteristics (e.g., generally low CCW scores in our Polish sample) or due to the different well-being measures used in the studies. That said, the robust correlations with psychopathology highlight the mental health relevance of CCW.

We also assessed the discriminant validity of the CCWS with anxiety and depression symptoms. Examining this type of validity is important in order to be sure that CCW represents a separable construct, which is statistically independent from an individual’s current level of general psychological distress. Our EFA extracted precisely two factors (i.e., a CCW factor with CCWS items and a psychological distress factor with PHQ-4 items), with strong factor loadings and no cross-loadings, thus supporting the discriminant validity of the CCWS, and suggesting that CCW can be distinguished from general levels of psychological distress.

### 4.3. Examination of the Underlying Structure of the Studied Variables

We were also interested in examining the underlying structure of our studied variables, and did this via a higher-order EFA. Our analysis extracted two distinct factors, with the first factor composed of climate-related variables and the second factor comprising general mental health variables. Overall, our analysis indicated that (1) CCW, the subjective experience of climate change, and environment protection behavior can form a coherent higher-order “active attitude towards climate change” factor, with CCW being a core element (i.e., having the strongest factor loadings), and that (2) this “active attitude towards climate change” factor was statistically separable from an individual’s current level of general psychological distress. The presence of a higher-order “active attitude towards climate change” factor comprising climate-related variables indicates that these variables are strongly interrelated. This empirical evidence indicates that the mutual influences of climate-related variables may be used as a basis for the development of psychological interventions to encourage different types of effective pro-environmental behaviors without maladaptive psychological responses on climate change.

Additionally, this analysis supported the idea that normal and moderate worry about climate change may be related to adaptive psychological responses, including paying attention to a threat in the environment, and behavioral engagement in environmental conservation efforts [3,24,25,26,27]. It should be stressed that this pattern was supported in our data-set, where CCWS scores were in general low, whereas subjective experience of climate change and behavioral engagement scores were high. It seems that this “active attitude towards climate change”, being only slightly positively associated with psychological distress, resulted from this special configuration of these climate-related variables with their specific levels (i.e., low CCWS scores, and high ECCS and BES scores). Some degree of worry could help people to seek and use information about pro-environmental behaviors effectively. Higher levels of worry, or even anxiety, may be overwhelming, and may not lead to adopting sustainable behaviors. We do not exclude that high CCWS levels (see for example, CCWS item 6: “I worry about climate change so much that I feel paralyzed in being able to do anything about it” [3]) would be associated with lower behavioral engagement. Therefore, in future studies, it seems beneficial to examine how people with different CCW levels implement behavioral engagement for environmental conservation efforts (e.g., via latent profile analysis).

### 4.4. Demographic Differences

In this study, we noted that females tended to have significantly higher CCWS scores than males, supporting previous reports [3]. No significant links between CCWS scores and age, education, and residence were noted. In our previous Polish study on the Climate Change Anxiety Scale [11], we noted similar results regarding gender differences, chiefly that females had higher levels of climate change anxiety than males. In the current study, we observed similar patterns regarding associations between CCW (CCWS scores) and age/education, as was found in work with the Slovenian CCWS [8]. Overall, our results consistently support previous findings that CCW seems to be higher in females [3]; therefore, gender should be included as a covariate in statistical analyses, whereas other studied demographic variables (i.e., age, education, and residence) seem to be less important in differentiating CCW levels.

Due to the relatively low number of CCWS validation studies, as well as differences in the demographics of their studied populations (e.g., age, gender, or cultural variation), the results derived from direct comparisons of CCWS scores (and their relationships with other variables) between different language versions of the CCWS should be considered tentative. In order to provide meaningful direct comparisons, future research with cross-cultural examinations of the measurement invariance of the CCWS will be of high value.

### 4.5. Practical Implications and Limitations of the Study

As this Polish version of the CCWS showed good psychometric properties, with confidence, it can be recommended for use in studies examining CCW. In order to facilitate its use, a copy of the questionnaire is freely provided in the Supplementary Materials. Being a brief self-report questionnaire, the Polish CCWS could be applied in a wide range of settings, and could be useful in developing the theory and practice of environmental psychology, as well as climate policy-making. For example, worry about climate change seems to be associated with climate policy support [28]; therefore, the assessment of CCW is of high importance in screening studies in general community samples across different countries around the world, especially among young people [4,29]. Identifying levels of CCW that can represent maladaptive psychological responses to climate change seems to be one of the tasks of clinical ecopsychology [30].

We feel this paper represents a useful contribution; however, there are several limitations of this study. First, this study was cross-sectional; thus, associations between the variables were bi-directional. This should be taken into account when interpreting the results. Second, our sample was relatively large; however, there were high proportions of females and younger adults. This may limit the generalizability of the obtained results. Third, due to a relatively small number of males, measurement invariance across gender was not evaluated. Fourth, our participants filled out the online versions of the questionnaires (except for the test–retest examination procedure); therefore, in future work it would be beneficial to also collect data using a paper-and-pencil method, and examine measurement invariance across paper-and-pencil and online versions of the CCWS. Fifth, the set of questionnaires we administered may have influenced the responses to the other questionnaires, so future research with different batteries will be useful to test the replicability of our findings. Last, our test–retest sample was moderate in size, and a larger sample for this purpose would be beneficial to future examinations of the temporal stability of CCWS scores and the CCW construct.

## 5. Conclusions

Overall, the Polish CCWS seems to have strong psychometric properties. The CCW construct is related to, but separable from, general psychological distress. CCW is also related to experiences of climate change and engagement in environmental protection behaviors. Future work with the CCWS should help to further explore the mental health consequences of climate change and the links between CCW and related climate change constructs.

## Figures and Tables

**Table 1:** Demographic characteristics of the participants.

Demographic Characteristics		*n*	%
Age (years)	Mean = 26.20, standard deviation (SD) = 10.61, minimum = 18, maximum = 70	420	100
Gender	Females	346	82.38
	Males	62	14.76
	Non-binary	12	2.86
Residence	Large cities (above 100,000 inhabitants)	199	47.38
	Towns (from 20,000 to 100,000)	71	16.90
	Small towns (up to 20,000)	55	13.10
	Villages	95	22.62
Education	University degree	107	25.48
	Secondary	280	66.67
	Vocational	12	2.86
	Primary	21	5.00

**Table 2:** Descriptive statistics of the CCWS items and standardized item factor loadings (all *p* < 0.001) from the CFA (*n* = 420).

CCWS Items	Mean	*SD*	Skewness	Kurtosis	Factor Loadings
1. “I worry about climate change more than other people.”	2.38	1.03	0.27	−0.74	0.76
2. “Thoughts about climate change cause me to have worries about what the future may hold.”	2.70	1.06	0.04	−0.65	0.84
3. “I tend to seek out information about climate change in the media (e.g., TV, newspapers, internet).”	1.94	0.95	0.75	−0.19	0.65
4. “I tend to worry when I hear about climate change, even when the effects of climate change may be some time away.”	2.54	1.14	0.20	−0.92	0.87
5. “I worry that outbreaks of severe weather may be the result of a changing climate.”	3.30	1.20	−0.47	−0.60	0.74
6. “I worry about climate change so much that I feel paralyzed in being able to do anything about it.”	1.45	0.80	1.96	3.70	0.48
7. “I worry that I might not be able to cope with climate change.”	2.12	1.14	0.67	−0.55	0.79
8. “I notice that I have been worrying about climate change.”	2.46	1.21	0.40	−0.81	0.85
9. “Once I begin to worry about climate change, I find it difficult to stop.”	1.66	0.87	1.28	1.20	0.75
10. “I worry about how climate change may affect the people I care about.”	2.46	1.28	0.43	−0.89	0.79

Note. All content of the CCWS items was reproduced from the original CCWS paper [3], with the permission of the developer of the CCWS, A. E. Stewart, who is one of the authors of the current article.

**Table 3:** Descriptive statistics and internal consistency reliability for the study variables.

Variables	Total Sample					Females			Males			Non-Binary		
	ω (95% CI)	α (95% CI)	*n*	Mean	*SD*	*n*	Mean	*SD*	*n*	Mean	*SD*	*n*	Mean	*SD*
CCWS Climate change worry	0.93 (0.93; 0.94)	0.93 (0.92; 0.94)	420	23.02	8.41	346	23.33	8.41	62	19.73	7.44	12	31.00	5.48
ECCS Experience of climate change	0.78 (0.73; 0.84)	0.77 (0.71; 0.83)	171	10.66	3.14	125	10.90	2.96	39	9.54	3.63	7	12.71	1.11
BES Behavioral engagement	0.73 (0.67; 0.79)	0.71 (0.64; 0.77)	171	24.92	3.79	125	25.67	3.34	39	22.38	4.31	7	25.57	2.51
PHQ-4 Anxiety	0.85 (0.80; 0.89)	0.85 (0.80; 0.89)	171	2.69	1.82	125	2.90	1.81	39	1.87	1.63	7	3.57	1.62
PHQ-4 Depression	0.85 (0.80; 0.89)	0.85 (0.80; 0.89)	171	2.57	1.92	125	2.67	1.90	39	2.21	1.89	7	2.71	2.29
PHQ-4 Total score	0.86 (0.81; 0.89)	0.85 (0.81; 0.89)	171	5.26	3.33	125	5.57	3.29	39	4.08	3.30	7	6.29	3.09
WHO-5 Well-being	0.82 (0.80; 0.85)	0.82 (0.80; 0.85)	410	8.76	4.37	338	8.55	4.38	60	10.03	4.43	12	8.25	2.26

**Table 4:** Pearson correlations between the study variables.

Variables	CCWS Climate Change Worry	ECCS Experience of Climate Change	BES Behavioral Engagement	PHQ-4 Anxiety	PHQ-4 Depression	PHQ-4 Total Score	WHO-5 Well-Being
CCWS Climate change worry	—						
ECCS Experience of climate change	0.59 ***	—					
BES Behavioral engagement	0.51 ***	0.52 ***	—				
PHQ-4 Anxiety	0.33 ***	0.14	0.11	—			
PHQ-4 Depression	0.20 **	0.04	−0.01	0.59 ***	—		
PHQ-4 Total score	0.30 ***	0.10	0.05	0.89 ***	0.90 ***	—	
WHO-5 Well-being	−0.02	−0.01	0.05	−0.43 ***	−0.56 ***	−0.55 ***	—

Note. ** *p* < 0.01; *** *p* < 0.001. Correlations between CCWS scores and WHO-5 scores are based on the sample of 410 people, whereas all other correlations between the study variables are based on the sample of 171 people.

**Table 5:** Factor loadings from the EFA of the CCWS items and PHQ-4 items (*n* = 171).

Variables	Factor 1 “CCW”	Factor 2 “General Psychological Distress”
CCWS item 1	0.75	
CCWS item 2	0.83	
CCWS item 3	0.67	
CCWS item 4	0.87	
CCWS item 5	0.75	
CCWS item 6	0.46	
CCWS item 7	0.77	
CCWS item 8	0.86	
CCWS item 9	0.74	
CCWS item 10	0.78	
PHQ-4 Anxiety item 1		0.70
PHQ-4 Anxiety item 2		0.76
PHQ-4 Depression item 1		0.89
PHQ-4 Depression item 2		0.71
Eigenvalues	6.56	2.42
The proportion of total variance for the rotated solution (%)	41.29	17.36

Note. Factor loadings lower than 0.20 are not displayed.

**Table 6:** Factor loadings from the second-order EFA of the study variables (*n* = 171).

Variables	Factor 1 “Active Attitude towards Climate Change”	Factor 2 “Negative Mental Health Outcomes”
CCWS Climate change worry	0.78	
ECCS Experience of climate change	0.75	
BES Behavioral engagement	0.68	
PHQ-4 Anxiety		0.68
PHQ-4 Depression		0.87
WHO-5 Well-being		−0.65
Eigenvalues	2.36	1.84
The proportion of total variance for the rotated solution (%)	28.01	27.66

Note. Factor loadings lower than 0.20 are not displayed.

## Data Availability

The raw data supporting the conclusions of this article will be made available by the authors on request.

## Supplementary Materials

The following supporting information can be downloaded at: https://www.mdpi.com/article/10.3390/healthcare12111128/s1, A copy of the Polish version of the Climate Change Worry Scale (CCWS).

## References

[1] Clayton S., Karazsia B.T. (2020). Development and validation of a measure of climate change anxiety. J. Environ. Psychol..

[2] Pihkala P. (2022). Toward a taxonomy of climate emotions. Front. Clim..

[3] Stewart A.E. (2021). Psychometric properties of the Climate Change Worry Scale. Int. J. Environ. Res. Public Health.

[4] Wullenkord M.C., Ojala M. (2023). Climate-change worry among two cohorts of late adolescents: Exploring macro and micro worries, coping, and relations to climate engagement, pessimism, and well-being. J. Environ. Psychol..

[5] van Valkengoed A.M., Steg L., de Jonge P. (2023). Climate anxiety: A research agenda inspired by emotion research. Emot. Rev..

[6] Marczak M., Wierzba M., Zaremba D., Kulesza M., Szczypiński J., Kossowski B., Budziszewska M., Michałowski J.M., Klöckner C.A., Marchewka A. (2023). Beyond climate anxiety: Development and validation of the inventory of climate emotions (ICE): A measure of multiple emotions experienced in relation to climate change. Glob. Environ. Chang..

[7] Ojala M., Cunsolo A., Ogunbode C.A., Middleton J. (2021). Anxiety, worry, and grief in a time of environmental and climate crisis: A narrative review. Annu. Rev. Environ. Resour..

[8] Plohl N., Mlakar I., Musil B., Smrke U. (2023). Measuring young individuals’ responses to climate change: Validation of the Slovenian versions of the climate anxiety scale and the climate change worry scale. Front. Psychol..

[9] Innocenti M., Santarelli G., Faggi V., Ciabini L., Castellini G., Galassi F., Ricca V. (2022). Psychometric properties of the Italian version of the climate change worry scale. J. Clim. Chang. Health.

[10] Wild D., Grove A., Martin M., Eremenco S., McElroy S., Verjee-Lorenz A., Erikson P. (2005). Principles of good practice for the translation and cultural adaptation process for Patient-Reported Outcomes (PRO) Measures: Report of the ISPOR Task Force for translation and cultural adaptation. Value Health.

[11] Larionow P., Sołtys M., Izdebski P., Mudło-Głagolska K., Golonka J., Rosińska M. (2022). Climate change anxiety assessment: The psychometric properties of the Polish version of the Climate Anxiety Scale. Front. Psychol..

[12] Kroenke K., Spitzer R.L., Williams J.B., Löwe B. (2009). An ultra-brief screening scale for anxiety and depression: The PHQ-4. Psychomatics.

[13] Larionow P., Mudło-Głagolska K. (2023). The Patient Health Questionnaire-4: Factor structure, measurement invariance, latent profile analysis of anxiety and depressive symptoms and screening results in Polish adults. Adv. Cogn. Psychol..

[14] World Health Organization, Regional Office for Europe (1998). Wellbeing Measures in Primary Health Care/the DepCare Project: Report on a WHO Meeting: Stockholm, Sweden, 12–13 February 1998.

[15] Cichoń E., Kiejna A., Kokoszka A., Gondek T., Rajba B., Lloyd C.E., Sartorius N. (2020). Validation of the Polish version of WHO-5 as a screening instrument for depression in adults with diabetes. Diabetes Res. Clin. Pract..

[16] Larionow P. (2023). Anxiety and depression screening among Polish adults in 2023: Depression levels are higher than in cancer patients. Psychiatria.

[17] Hu L., Bentler P.M. (1999). Cutoff criteria for fit indexes in covariance structure analysis: Conventional criteria versus new alternatives. Struct. Equ. Model..

[18] McDonald R.P. (1999). Test Theory: A Unified Treatment.

[19] Cronbach L.J. (1951). Coefficient alpha and the internal structure of tests. Psychometrika.

[20] Groth-Marnat G. (2009). Handbook of Psychological Assessment.

[21] Koo T.K., Li M.Y. (2016). A guideline of selecting and reporting intraclass correlation coefficients for reliability research. J. Chiropr. Med..

[22] Lovakov A., Agadullina E.R. (2021). Empirically derived guidelines for effect size interpretation in social psychology. Eur. J. Soc. Psychol..

[23] Brown T.A. (2006). Confirmatory Factor Analysis for Applied Research.

[24] Berry H.L., Peel D. (2015). Worrying about climate change: Is it responsible to promote public debate?. BJPsych Int..

[25] Verplanken B., Marks E., Dobromir A.I. (2020). On the nature of eco-anxiety: How constructive or unconstructive is habitual worry about global warming?. J. Environ. Psychol..

[26] Verplanken B., Roy D. (2013). “My worries are rational, climate change is not”: Habitual ecological worrying is an adaptive response. PLoS ONE.

[27] Sampaio F., Costa T., Teixeira-Santos L., de Pinho L.G., Sequeira C., Luís S., Loureiro A., Soro J.C., Roldán Merino J., Moreno Poyato A. (2023). Validating a measure for eco-anxiety in Portuguese young adults and exploring its associations with environmental action. BMC Public Health.

[28] Bouman T., Verschoor M., Albers C.J., Böhm G., Fisher S.D., Poortinga W., Whitmarsh L., Steg L. (2020). When worry about climate change leads to climate action: How values, worry and personal responsibility relate to various climate actions. Glob. Environ. Chang..

[29] Wu J., Snell G., Samji H. (2020). Climate anxiety in young people: A call to action. Lancet Planet. Health.

[30] Thoma M.V., Rohleder N., Rohner S.L. (2021). Clinical ecopsychology: The mental health impacts and underlying pathways of the climate and environmental crisis. Front. Psychiatry.

