# Supplementary material for: The Climate Change Worry Scale (CCWS) and Its Links with Demographics and Mental Health Outcomes in a Polish Sample
Source: Healthcare (Basel). 2024 May 31;12(11):1128. doi: 10.3390/healthcare12111128 (PMC11171471; doi:10.3390/healthcare12111128)
Supplement: Supplementary file 1 [file healthcare-12-01128-s001.zip › healthcare-2995152-supplementary.pdf]

A copy of the Polish version of the Climate Change Worry Scale (CCWS)
Skala zaniepokojenia zmianami klimatu

Instrukcja. Proszę przeczytaj każde stwierdzenie i wskaż, jak często odnosi się ono do Ciebie. Odpowiedz (zaznacz kółkiem), jak się zazwyczaj czujesz. Nie ma dobrych ani złych odpowiedzi.

		Nigdy	Rzadko	Czasami	Często	Zawsze
1	Martwię się zmianami klimatu bardziej niż inni ludzie.	1	2	3	4	5
2	Myśli o zmianach klimatu powodują, że zaczynam martwić się tym, co może przynieść przyszłość.	1	2	3	4	5
3	Mam tendencję do wyszukiwania informacji na temat zmian klimatu w mediach (np. w telewizji, gazetach, Internecie).	1	2	3	4	5
4	Mam tendencję do martwienia się, gdy słyszę o zmianach klimatu, nawet kiedy ich skutki mogą wystąpić w przyszłości.	1	2	3	4	5
5	Obawiam się, że występowanie ekstremalnych zjawisk pogodowych może być skutkiem zmieniającego się klimatu.	1	2	3	4	5
6	Martwię się zmianami klimatu tak bardzo, że czuję się sparaliżowany/a w podejmowaniu działań prośrodowiskowych.	1	2	3	4	5
7	Martwię się, że może nie będę w stanie poradzić sobie ze zmianami klimatu.	1	2	3	4	5
8	Dostrzegam to, że martwię się zmianami klimatu.	1	2	3	4	5
9	Kiedy zaczynam się martwić zmianami klimatu, to trudno mi przestać.	1	2	3	4	5
10	Martwię się o to, jak zmiany klimatu mogą wpłynąć na osoby, na których mi zależy.	1	2	3	4	5

Skala zaniepokojenia zmianami klimatu (*Climate Change Worry Scale*; CCWS) jest 10-pozycyjnym samoopisowym kwestionariuszem do badania niepokoju związanego ze zmianami klimatu. Skala jest 1-czynnikowa. Żeby obliczyć wynik CCWS, należy zsumować wyniki wszystkich pozycji. Wyższe wyniki oznaczają wyższe nasilenie niepokoju związanego ze zmianami klimatu.
